# Giant lipoma of the left mesocolon: Radiological and surgical aspects

**DOI:** 10.1016/j.ijscr.2019.04.029

**Published:** 2019-04-19

**Authors:** Adjirata Koama, Nayi Zongo, Nina Astrid Nde/Ouédraogo, Benilde Marie Ange Kambou/Tiemtoré, Olga Melanie Lompo, Adama Sanou, Ouséni Diallo, Claudine Lougué/Sorgho, Rabiou Cissé

**Affiliations:** a Radiology and Medical Imaging Unit, University Hospital Centre of Bogodogo, Ouagadougou, Burkina Faso; b Visceral Surgery at Yalgado Ouedraogo University Hospital Centre (CHUYO), Burkina Faso; c Visceral Surgery at Blaise Compaoré National Hospital (HNBC), Burkina Faso; d Pathological Anatomy, CHUYO, Ouagadougou, Burkina Faso; e Radiology and Medical Imaging Unit, CHUYO, Ouagadougou, Burkina Faso

**Keywords:** Lipoma, Left mesocolon, Scan, MRI, TEP scan, Hemicolectomy

## Abstract

**Introduction:**

Abdominal masses are common in digestive surgery and gastro-enterology units. However, meso-intestinal lipomas remain rare and lipoma of the left colon uncommon. We report a case of giant lipoma of the left mesocolon whose diagnosis was highly guided by radiological examinations.

**Presentation of case:**

A female patient aged 56, consulted for left subcostal abdominal pains. The clinical examination showed an abdominal mass occupying the left hemiabdomen. The abdominal-pelvic CT scan highlighted a large abdominal-pelvic mass in the left abdomen. Abdominal-pelvic MRI revealed a large fatty mass spreading from the front subphrenic space up to the level of the left iliac fossa, non-suspected and compatible with lipoma. FDG-Pet Scan had not revealed pathological fixing. The mass appeared like a total gap space. Exploratory surgery revealed a lipoma mass in the left mesocolon. Hemicolectomy was performed taking away the mass. Histology confirmed the diagnosis of lipoma and the outcome was favourable.

**Discussion:**

Our case represents the fourth case of mesocolon lipoma described in the literature. Imaging, especially TDM and MRI are an important step of the preoperative diagnosis. The surgery consists of either a lumpectomy or a colectomy.

**Conclusion:**

Lipoma of the left mesocolon is exceptional. Radiological examinations provide most arguments to suggest lipoma. However the organ’s diagnosis is provided by surgical exploration and the certainty diagnosis by pathological examination. Treatment is surgical.

## Highlights

• Epidemiology: Lipoma of the mesocolon is a rare tumour less described in the literature.
• Diagnosis: It generally involves large masses. Clinically, it is often asymptomatic. When they exist, these symptoms are less specific and generally due to the large size of the tumour (compression, invagination, hernia). Imaging, especially TDM and MRI are an important step of the preoperative diagnosis. In imaging as in anatomopathology, lipoma-like liposarcoma is the main differential diagnosis. A differential diagnosis with lipoma-like sarcoma must be done.
• Treatment: Treatment is surgical. However, there are variations in the surgical procedures. Some authors had carried out lumpectomy. In our case, we carried out a left colectomy removing the tumour and the mesocolon, as well as the satellite lymph nodes. Each approach has its arguments, i.e. a conserving treatment exposing to repetition if the histological and/or immunochemistry data come out less reassuring, and a more secure and less invasive treatment. There is no consensus on the procedure which depends on the teams.

## 1 Introduction

Lipomas are benign mesenchymal tumours that develop at the expense of fat tissue [[1], [2], [3]]. They are commonly described as being subcutaneous [1,3]. When intraabdominal, they locate in the gastrointestinal tract and in the retroperitoneum [[1], [2], [3]]. Lipoma of the mesocolon is a rare entity. On the basis of a research not limited in time, only three cases have been described to date. The first by Pétrin in 1993 [4], the second by Versaci in 2006 [5] and the third by Pachini in 2011 [1]. We report the fourth case, insisting on the radiological and therapeutic aspects.

The management of this case as well as literature search was performed and the work has been reported in line with the SCARE criteria [6].

## 2 Presentation of case

**Fig. 1:**
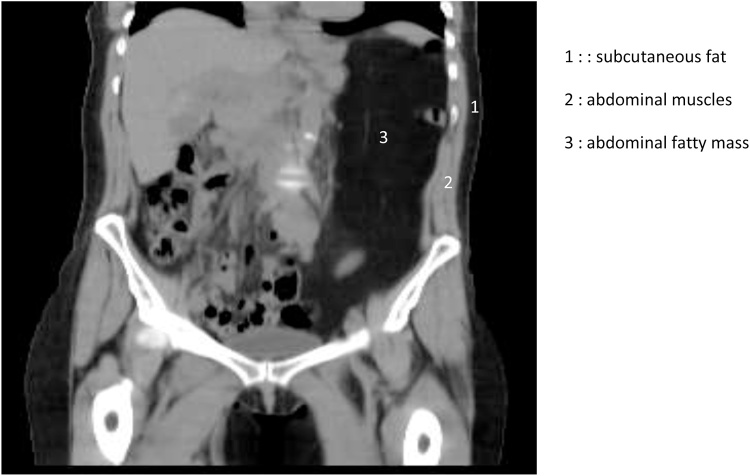
Coronal cut showing the lipomatous lass of the left mesocolon (TDM).

**Fig. 2:**
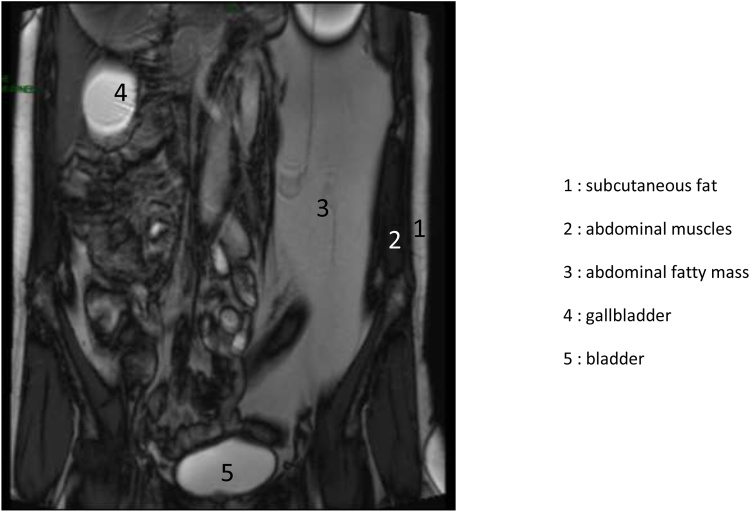
Coronal cut showing the lipomatous lass of the left mesocolon (MRI) in T2 hypersignal.

Patient, aged 56 years, mother of five children consulted for left subcostal pains for several months. The pains were more intense in a sitting position. These pains were followed by episodes of nausea/vomiting and even a subocclusive syndrome. She also mentioned rare and episodic rectal bleeding. The examination revealed an abdominal mass covering all the left hemiabdomen, from the left costal edge and the xiphoid process to the pubis. Upon palpation, it was somewhat painful, less mobile and soft. It measured about 28 cm long and 15 cm wide. Colonoscopy done was normal. The scan revealed a fatty mass in the left hypochondrium, similar to a fatty subcutaneous tissue (Fig. 1). It was homogeneous, well-defined and extended from the left diaphragmatic cupola to the pelvis, covering almost all the left hemiabdomen. The abdominal-pelvic MRI also showed this homogeneous fatty mass similar to a subcutaneous fat mass in T1 and T2 hypersignal (Fig. 2). It had clear contours and extended from the left diaphragmatic cupola to the pelvis, touching the left kidney all along its front side and lateral edge. It looked like mesenteric lipoma without argument for degenerescence. FDG Pet Scan had not demonstrated pathological fixing.

The mass appeared like a total gap space.

We performed left hemicolectomy to remove in one piece the mass with the descending colon. The inferior mesenteric artery is not attached to its root. We attached its branches (upper left colonic artery and descending colon artery). Cutting of transverse colon and sigmoid colon was done after a colo-parietal detachment and release of the left colonic angle. Restoration of continuity was done through colo-colonic termino-terminal anastomosis by 2 hemisurjets with prolene 4/0. The mesocolonic breach was also closed with Prolene 4/0.

Macroscopic examination revealed an adipose tumour of 39 cm located in the mesocolon and whose histology did not show any sign of malignity. A full exeresis was carried out. Given its size and location, an opinion was requested from the Sarcoma Expertise Centre, which helped exclude the hypothesis of well-differentiated lipoma-like liposarcoma. Postoperative follow-ups were uneventful. The patient’s recovered his bowel motility on the third day. He left hospital on the fifth day. After a period of 8 years, he made no complaint and no recurrence was noticed.

## 3 Discussion

Lipomas are benign connective tumours which develop at the expense of fat tissue [1,2,7]. They grow slowly and form round soft tissue masses surrounded or not by a thin fibre capsule [1,7,8]. It is the most common soft tissue tumour [1,8] and also the most frequent adipose tumour according the 2013 classification by the World Health Organization [2]. Its superficial subcutaneous localization on the trunk, thigh, forearm and nape of the neck is well described in the literature [1,2]. Although less frequent, it may localize in any fatty tissue space, may be intramuscular, intermuscular, in the retroperitoneum or in the gastro-intestinal tract [1,2,9]. Its localization in the mesocolon was exceptionally described in the literature by Pétrin in 1993 [4], Versaci in 2006 in the transverse mesocolon and Pachani in 2011 in the meso-sigmoid [1,5]. We report the fourth observation in the literature. These tumours may occur at any age, but the prime age is between the fourth and seventh decade [1,2,8]. A male predominance was reported concerning common localization lipoma [2,3]. As for lipoma of the mesocolon, Pachania observed it in a male patient aged 68 years [1], and we observed it in a female patient of 53 years. As this localization is scarce, a specific prime age or sex predominance cannot be defined.

Diagnosis is based on clinical, radiological and histological examinations [1,3,8]. This tumour is often clinically silent, when it is superficially located and causes at most aesthetic effects [1]. Typically, it has a size inferior to 10 cm [1,3]. When intraabdominal it is often large [1,3,4] and may be noisy. As clinical presentation, we move from the absence of symptoms to the surgical table of acute occlusion of invagination [1,4,7]. Non-specific abdominal pains, a palpable abdominal mass, gastro-intestinal bleeding, nausea and vomiting were described in the literature [1,4] like for our patient. The WHO Soft Tissue Tumour Classification Committee divides benign lipoma tumours into nine groups based on histology, depending on whether the lesion is fully composed of mature adipose tissue, is intimately associated with non-adipose tissue or is made up of brown fat [2,3]. The classification proposed by Weiss and Goldblum seems to be of great clinical and radiological use according to Bancroft [3]. It distinguishes five major categories including sub-categories for some of them [3]. Radiological features are often similar in each category opening door for some differential radiological diagnoses [3]. Radiology, namely tomodensitometry (TDM) and Magnetic Resonance Imaging (MRI) of adipose masses are sufficiently distinguishing to suggest a specific diagnosis [3,6,7]. At TDM, lipoma in its typical form appears as a fatty mass similar to subcutaneous fat, well-defined and not-enhanced after injection of contrast product in arterial and portal phases. It may contain thin spans slightly enhanced after injection of contrast product [3,7]. Calcifications are observed in 11% of cases [3]. This distinguishing feature was observed in our case and strongly guided the diagnosis. At scanning, subcutaneous fat in the abdominal wall had the same aspect as intraabdominal mass of the left flank. At MRI, the adipose mass appeared as a well-limited homogeneous fatty mass comparable to the signal of subcutaneous fat tissue, i.e. in T1 and T2 hypersignal and loss of signal (without signal) on sequences with suppression of fat signal, T2 Fat Satet STIR [3,6,7]. These data are similar to what we noticed in our observation. No tissue portion or microcalcification was observed in our case. In imaging, the association of such fatty mass with a dense or different signal portion tissue leads to the tumour being classified in one of the 4 other types of adipose tumours according to the Weiss and Goldblum Classification, depending on the features of this portion [3]. However, when a mass does not meet the diagnosis criteria of a lipoma, especially irregularity, heterogeneity, the presence of thick and irregular nodular or interrupted septa, and nodular components of soft or non-adipose tissue, a diagnosis suspecting liposarcoma must be suggested [3,8], even though it is most often a diagnosis of exclusion [3]. At MRI, in addition to the above-mentioned features, the fact that there is incomplete suppression of fat with linear or nodular hyperdense areas on T2 FATSAT and STIR sequences also suggest the diagnosis of liposarcoma [3,6,8]. Atypical forms of lipoma are numerous; only histology and in some cases immunochemistry [2,3,5] can help establish an accurate diagnosis. The obsession of both the radiologist and the anatomopathologist is the lack of clear knowledge of liposarcoma, especially “lipoma-like” liposarcoma [2,3,10,11]. Amato in 1998 had reported the 4th case of liposarcoma of the mesocolon. It was liposarcoma “looking like lipoma” well differentiated from the sigmoid mesocolon [1,10]. A differential diagnosis with lipoma-like sarcoma was made thanks to a review of blades in a Sarcoma Expertise Centre. Whether it is malignant or benign, the treatment of adipose tumour of the mesocolon is unanimously surgical in the literature [1,5,10,11]. However, there are variations in the surgical procedures. The team of Pachani had carried out lumpectomy and fixing of the descending colon [1]. In our case, we carried out a left colectomy removing the tumour and the mesocolon, as well as the satellite lymph nodes. Each approach has its arguments, i.e. a conserving treatment exposing to repetition if the histological and/or immunochemistry data come out less reassuring, and a more secure and less invasive treatment. There is no consensus on the procedure which depends on the teams.

## 4 Conclusion

Lipoma of the mesocolon is a rare tumour less described in the literature. It generally involves large masses. Clinically, it is often asymptomatic. When they exist, these symptoms are less specific and generally due to the large size of the tumour (compression, invagination, hernia). Imaging, especially TDM and MRI are an important step of the preoperative diagnosis. In imaging as in anatomopathology, lipoma-like liposarcoma is the main differential diagnosis. Treatment is surgical, but procedures depend on the teams. Our case represents the fourth case of mesocolon lipoma described in the literature. Its dignostic and therapeutic procedures must be known signifies its importance for reporting in the English literature.

## Conflicts of interest

The authors declare that they have no competing interests regarding the publication of this manuscript.

## Sources of funding

No sponsors to declare.

## Ethical approval

Ethical approval is not needed for this case report as patient consent and we are not trialing a new device.

## Consent

Written and signed consent by the patient to publish a case report has been obtained.

## Author’s contribution

Case report concept and design: Koama A, Zongo N, Nde/Ouédraogo NA.

Acquisition of data: Koama A, Zongo N, Kambou/Tiemtoré B, Lompo OM.

Statistical analysis and interpretation of data: Koama A, Sanou A, Diallo O, Lougué/Sorgho C, Cissé R.

Drafting of the manuscript: Koama A, Sanou A, Lougué /Sorgho C, Cissé R, Houry S.

Critcal revision of the manuscript for important intellectual content: Koama A, Sanou A, Diallo O, Lougué /Sorgho C, Cissé R.

All authors approved the final version of this publication.

## Registration of research studies

It is not a clinical trial.

## Guarantor

Dr Adjirta Koama.

Dr Nayi Zongo.

## Provenance and peer review

Not commissioned, externally peer-reviewed.
